# Digital inequities and emotional resilience: understanding the impact of the COVID-19 pandemic on preclinical medical students

**DOI:** 10.3389/fmed.2025.1559536

**Published:** 2025-06-12

**Authors:** William A. H. Schwartz, Tazim S. Merchant, Isi Sturla, Leah C. Neubauer

**Affiliations:** ^1^Department of Psychiatry, Lenox Hill Hospital, New York, NY, United States; ^2^Department of Obstetrics and Gynecology, Mayo Clinic, Rochester, MN, United States; ^3^The Institute for Family Health, New York, NY, United States; ^4^Department of Health Behavior and Health Equity, School of Public Health, University of Michigan, Ann Arbor, MI, United States

**Keywords:** medical education, student mental health, digitization, hybrid learning, educational inequities, isolation

## Abstract

**Introduction:**

The COVID-19 pandemic profoundly disrupted medical education, posing unique challenges for first-year medical students transitioning into preclinical training. Traditional in-person instruction was replaced with virtual and hybrid learning, creating barriers related to digital access, social isolation, academic engagement, and mental health. This study explores the academic, social, and emotional impacts of these disruptions to identify actionable strategies for fostering resilience and inclusivity in medical education systems.

**Methods:**

A pragmatic research framework guided a student-led needs assessment via a survey distributed to first-year medical students during the 2020–2021 academic year. The survey included demographic questions and open-ended prompts about academic, social, and emotional experiences. Data from 57 respondents (36% response rate) were analyzed thematically using the constant comparative method, and findings were validated through member-checking.

**Results:**

Five key themes emerged: isolation, difficulty engaging with virtual curricula, lack of community, mental health challenges, and perceived convenience of virtual learning. Isolation was the most prominent theme, with students reporting emotional distress and limited peer connections. Engagement difficulties stemmed from screen fatigue, reduced accountability, and blurred personal-academic boundaries. Mental health concerns, including anxiety and emotional exhaustion, were widespread, although some students cited protective coping strategies such as structured routines. A small subset highlighted benefits of virtual learning flexibility.

**Discussion:**

These findings underscore the need for hybrid curricular models that balance digital flexibility with structured opportunities for peer interaction, enhanced mental health services, and sustained institutional support. Addressing these challenges is critical for building equitable and resilient medical education systems prepared for future crises.

## Introduction

The COVID-19 pandemic triggered unprecedented disruptions in education systems worldwide, amplifying existing inequalities and revealing significant vulnerabilities in access to quality education. Medical education, a field deeply reliant on hands-on, in-person instruction and mentorship, faced particular challenges in maintaining continuity and ensuring equitable opportunities for all students. As institutions transitioned to virtual and hybrid learning environments, traditional pedagogies were replaced with pre-recorded lectures, online discussions, and remote simulations (1–3). While digitalization offered opportunities for continuity, it also introduced new barriers related to technological access, digital literacy, and the social dimensions of learning.

The existing literature extensively documents curricular adaptations during the pandemic, with emphasis on clinical training and the role of student-led grassroots efforts in securing access to learning opportunities (4, 5). Perspectives from medical school administrators have also been explored, primarily focusing on logistical and operational challenges during the transition (6). However, existing research has shown limited attention to the nuanced experiences of preclinical medical students, who navigated foundational academic and social transitions under unprecedented conditions. Existing accounts, such as Theoret and Ming’s focus on clinical training disruptions or Jacobson’s anecdotal reflections on distancing restrictions, highlight key challenges but fall short of providing a comprehensive analysis of students’ academic, emotional, and social well-being during this period (7, 8).

Understanding the intersection of crisis-driven digitalization, educational inequalities, and student well-being is critical for creating resilient education systems. Preclinical students faced unique relational, social, and cognitive challenges that extend beyond curricular concerns (9). As hybrid learning models become an enduring feature of education, addressing these dimensions is essential to safeguard equitable access to quality education, foster inclusivity, and mitigate the long-term impacts of educational disruptions.

This study presents findings from a student-led needs assessment involving first-year medical students during the 2020–2021 academic year, based on a qualitative analysis of open-ended responses collected as part of a broader continuing medical education (CME) program evaluation. Using a pragmatic approach grounded in real-world educational challenges, this analysis draws on the constant comparative method and thematic analysis to explore students’ academic, social, and emotional experiences during their transition to medical school amidst the COVID-19 pandemic. The goal is to generate actionable insights that support the development of more inclusive and resilient medical education systems in the face of ongoing and future disruptions.

## Methods

This study employed a pragmatic, student-led qualitative approach to explore and understand the multifaceted impacts of the COVID-19 pandemic on medical students’ experiences during their transition to medical school. Pragmatism emphasizes flexibility, real-world applicability, and outcome-driven inquiry, making it particularly valuable for investigating complex social phenomena such as pandemic-driven disruptions in education (10). This study represents a secondary analysis of data originally collected as part of a Continuing Medical Education (CME) intervention evaluation survey. While the dataset was primarily gathered for program evaluation, its richness enabled a focused qualitative analysis on the psychosocial, cognitive, and emotional impacts of pandemic learning on first-year medical students. Secondary analyses of existing datasets are widely recognized as valuable for extracting deeper insights, provided the analysis aligns with the dataset’s original intent and methodological framework (11).

The data were collected using a Google Form survey, which included both demographic and qualitative components. The primary open-ended question asked participants to describe how COVID-19 impacted their medical education, with prompts to consider aspects such as studying, social connections, and mental health (Table 1). Demographic data were also collected, including gender, sex at birth, race, age range, first-generation college status, and Pell Grant eligibility. The inclusion of Pell Grant eligibility, a U.S. Federal financial aid program for undergraduate students demonstrating financial need, served as a proxy for socioeconomic status. This variable offered context for potential disparities in student experiences during the pandemic, acknowledging that financial strain might influence students’ ability to adapt to remote learning and other pandemic-related challenges.

**Table 1:** Full survey questionnaire for student respondents to evaluate continuing medical education (CME) intervention.

Section	Question #	Question stem	Answer type
Demographics	1	“What sex were you born as?”	Multiple-choice response
	2	“To which gender do you most closely identify?”	Multiple-choice response
	3	“To which race do you most closely identify?”	Multiple-choice response
	4	“Are you of Hispanic, Latinx, or Spanish origin?”	Multiple-choice response
	5	“Which age category do you fall into?”	Multiple-choice response
	6	“Are you a first-generation college student?”	Multiple-choice response
	7	“Are you Pell Grant eligible?”	Multiple-choice response
Intervention	8	“When did you start meeting with your study group?”	Forced-choice response
	9	“Did you meet with your study group virtually or in person?”	Forced-choice response
	10	“If both, how many meetings were in person? (Skip if not applicable)”	Forced-choice response
	11	“How often did you meet your study group?”	Forced-choice response
	12	“What group were you assigned to?”	Forced-choice response
	13	“Did that group structure change?”	Forced-choice response
	14	“If your group structure did change, what structure did it change to? (Skip if not applicable)”	Forced-choice response
	15	“What is the approximate duration of your study group meetings?”	Forced-choice response
	16	“How long, on average, did you spend preparing for study group?”	Forced-choice response
Impact	17	“Rate how much you agree with the following statements (1 = strongly agree, 2 = agree, 3 = neutral, 4 = disagree, 5 = strongly disagree)”	
	17a	“The COVID-19 pandemic influenced my decision to join a study group.”	Likert-response scale
	17b	“I participated in a study group because medical education was virtual.”	Likert-response scale
	17c	“My study group(s) provided me with emotional support”	Likert-response scale
	17d	“I felt comfortable participating in my study group”	Likert-response scale
	17e	“The social interaction with my peers has helped me with my learning”	Likert-response scale
	17f	“I have made new friends as a result of my study group”	Likert-response scale
	17 g	“Study group preparation motivated me to study more than I would have on my own.”	Likert-response scale
	17 h	“Study groups were well-organized.”	Likert-response scale
	17i	“Being in a study group showed me where I stood in comparison to other students with regard to my depth of knowledge”	Likert-response scale
	17j	“My study group helped me prepare for the module exam”	Likert-response scale
	18	“Describe how COVID-19 impacted your medical education (e.g., how you study, how you meet people, your mental health, etc.)”	Open-response
	19	“Describe what role you feel the voluntary study groups played in addressing COVID-19 impacts on your experience as a medical student?”	Open-response
	20	“Were there other programs, similar or different to this one, that eased your transition to medical school during the pandemic?”	Open-response
	21	“What did you enjoy most about the study groups?”	Open-response
	22	“Describe the most useful aspects of the study groups.”	Open-response
	23	“Describe the most challenging aspects of study groups.”	Open-response
	24	“Have you continued in study group after the Foundations I exam?”	Forced-choice response
	25	“If so, why? If not, why not?”	Open-response
	26	“Are there any other programs you felt would have been more beneficial than study groups in easing your transition to medical school during COVID? If so, what are they?”	Open-response
	27	“Please share anything else about the study group experience here:”	Open-response

This study received IRB-exempt status (STU00213851) under the category of program evaluation, and per institutional policy, no additional IRB review was required for the secondary analysis and publication of de-identified, aggregate data derived from the same dataset. All data used in this secondary analysis were fully de-identified prior to review, and steps were taken to preserve participant confidentiality, including the removal of any potentially identifying language from quoted material. Participants were eligible if they had attended at least one CME intervention session, and to incentivize participation, 10 randomly selected students received gift cards or school apparel.

Survey dissemination followed a multi-channel strategy to maximize participation. Surveys were distributed through school email listservs, class-specific Facebook groups, and individualized outreach by the study authors. Weekly reminders were sent using both email and social media platforms to encourage participation and ensure adequate response rates. The survey remained open for 29 days, providing students ample time to participate despite their academic schedules and other responsibilities.

Data analysis combined the constant comparative method with thematic analysis, integrating the strengths of both approaches to ensure methodological rigor and meaningful insight extraction. The constant comparative method, originally developed within Grounded Theory by Glaser and Strauss (12), involves iterative comparison of emerging data with previously analyzed data to refine themes and identify patterns. This method ensured that recurring ideas were systematically identified and refined throughout the analytical process. Thematic analysis, as described by Braun and Clarke (13), provided a structured approach for identifying, analyzing, and synthesizing patterns across the dataset. Its flexibility allowed the researchers to balance inductive theme generation with alignment to the study’s research objectives, ensuring that emergent findings remained anchored in the data. The combination of these two methodologies offered both depth and breadth in analysis, enabling the research team to extract nuanced insights while maintaining systematic consistency across the coding and thematic synthesis.

The analytical process began with three student authors independently coding the responses to identify recurring patterns and initial themes. After the initial coding phase, the team collaboratively developed a codebook, refining definitions for each theme and resolving discrepancies through group discussion. A single author then re-coded the entire dataset using the finalized codebook to ensure internal consistency and alignment with the agreed-upon thematic framework. This process was complemented by the role of a ‘critical friend,’ a faculty advisor who provided external reflection and constructive critique throughout the analysis. As described by Costa and Kallick (14), the critical friend role involves constructive questioning and reflective dialog, which serves to challenge assumptions, refine analytical interpretations, and enhance the study’s overall methodological rigor.

To further validate the findings, a member-checking process was conducted using a follow-up Google Form. This form listed the five key insights derived from the analysis and asked participants whether they agreed with the identified themes. Respondents could select “Yes,” “No,” or “Other,” and free-text fields allowed them to suggest additional themes or clarify existing ones. Importantly, this member check included students who had not participated in the original survey, further ensuring that the findings represented diverse perspectives within the student cohort. Member checking provided an essential opportunity to verify the accuracy of the identified themes and to incorporate additional insights from participants.

The choice to integrate constant comparative analysis with thematic analysis was intentional. Pragmatism allowed the researchers to adapt their approach to the complexity of the research question, while the constant comparative method ensured systematic analysis of the data. Thematic analysis provided a structured yet flexible lens for identifying patterns and drawing meaningful conclusions. These methodological choices align with the study’s goal of producing findings that are both methodologically rigorous and practically actionable for medical education leadership. Together, these approaches created a robust analytical foundation for understanding the psychosocial, cognitive, and emotional impacts of COVID-19 on the transition into medical school, ensuring that the study contributes both to scholarly literature and to institutional decision-making processes.

## Results

A total of 57 responses were received, representing approximately 36% of the medical school class (57/160) and 40% (57/146) of students who signed up for the CME intervention. These responses provided a rich dataset for thematic analysis, allowing for the identification of key themes that captured both common experiences and underrepresented perspectives among students.

### Demographics

The participant pool included a slight majority of cisgender women (*n* = 33; 57.9%). Students identified across multiple racial and ethnic groups, with Asian students comprising the largest group (56.1%), followed by white (31.6%), Black (7.0%), and multiracial students (5.3%). The majority of respondents were between the ages of 20 and 24 (80.7%). Only two participants (3.5%) reported being Pell Grant eligible, indicating a small representation of students from federally recognized low-income backgrounds (Table 2).

**Table 2:** Demographic data from medical student respondents in 2020–2021 academic year (*n* = 57).

Characteristic	Frequency (*n*= 57)	Percentage
Gender		
Cisgender women	33	57.89%
Cisgender men	23	40.35%
Preferred not to answer	1	1.75%
Ethnicity		
Hispanic or Latino	6	10.52%
Race		
Asian	32	56.14%
White	18	31.57%
Black	4	7.01%
Multiracial	3	5.26%
Age		
20–24	46	80.70%
25–29	10	17.54%
30+	1	1.75%
Pell grant eligibility	2	3.51%

### Overview of themes

Thematic analysis revealed five key themes, with four reflecting challenges faced by the majority of respondents—Isolation, Difficulty Engaging, Lack of Community, and Mental Health—and one highlighting a benefit noted by a small subset of participants (Convenience) (Table 3). Member-checking, which included 51 respondents (approximately 32% of the class), confirmed the validity of these themes, with 98% agreement on their representativeness. Notably, 8 respondents (16%) who had not participated in the original survey contributed their perspectives during the member-checking process, further broadening the dataset’s reach.

**Table 3:** Thematic analysis of “Describe how COVID-19 impacted your medical education,” answered by medical students.

Key themes	Description	Student responses
Isolation	Students spending more time alone and meeting less people than one would have leading to feelings of emotional loneliness.	51 (89.47%)
Difficulty engaging	Academic content is more difficult to pay attention to, enthusiasm is decreased, less motivation and focus, increased screen time fatigue	18 (31.57%)
Lack of community	Lack of meaningful relationships and support system among students	14 (24.56%)
Mental health	Negative impact on student mental health (i.e., sadness, anxiety, loneliness, etc.)	11 (19.0%)
Convenience	COVID-19 pandemic promoted academic environments suited to certain students (flexibility, motivation, efficiency, personality, etc.)	4 (7.01%)

#### Isolation

Isolation emerged as a dominant theme, with 89.5% (*n* = 51) of respondents discussing how pandemic-related restrictions heightened their sense of social and emotional isolation. Academic and emotional impacts were deeply intertwined. Students described a lack of informal social interactions, which previously served as both a source of emotional support and a means to share academic insights. One student remarked, “One of the biggest impacts of COVID-19 has been the inability to meet people. I feel as though I have struggled, especially living alone, meeting other M1s.” This absence of peer connection not only caused emotional distress but also disrupted informal academic exchanges that often occur in casual social settings, such as libraries or study lounges.

The emotional toll of isolation was evident in students’ descriptions of their daily routines. One participant shared, “There were days when I would not speak to anyone at all. Zoom makes it so hard to have casual conversations—it feels so formal all the time.” These accounts suggest that beyond structured curricular opportunities, unstructured peer interactions play an essential role in fostering both academic motivation and emotional well-being.

To cope with isolation, students often relied on existing support systems from undergraduate friendships, family, or previously established social networks, rather than building new relationships within their medical school cohort. Others found limited solace in structured curricular mechanisms, such as study groups or virtual small-group sessions. One student described, “I met people only through structured means, such as [my] college, my apartment building, or study group.”

Academically, isolation also forced changes in study habits. Students who thrived in social study environments struggled to adapt to remote, independent studying. One respondent stated, “As someone who definitely benefits from social studying, the pandemic made me change my habits. I now study on my own a lot more, but I’ve found ways to set up virtual study groups to mimic what I was missing.” These findings underscore the dual academic and emotional consequences of isolation, emphasizing the importance of addressing both dimensions in future curricular adaptations.

#### Difficulty engaging

A notable subset of participants (31.6%, *n* = 18) reported significant difficulties engaging with virtual curricula. Students described persistent screen fatigue, a lack of focus, and reduced enthusiasm for learning. One participant remarked, “Staring at a computer screen all day at my desk makes it incredibly hard for me to concentrate on anything.” Another shared, “The virtual environment takes away my motivation to study because I do not really feel like I’m in school.”

For many, the lack of physical separation between home and academic spaces further compounded these difficulties. The blurring of boundaries disrupted their ability to remain focused during lectures or discussions. Some students also noted that the absence of real-time peer interaction in virtual classrooms diminished the sense of shared purpose and accountability typically fostered by in-person learning environments. These narratives highlight how the shift to virtual platforms posed challenges not only to academic focus but also to intrinsic motivation and learning engagement.

#### Lack of community

Approximately 24.6% (*n* = 14) of respondents articulated a deep sense of disconnection from their peers and the larger medical school community. Many students emphasized that while they knew their peers on a surface level, they lacked meaningful connections or support networks. One respondent explained, “Starting medical school is stressful enough as a transition; compounding it with a global pandemic made it much more difficult to build a community, find a support network, and have honest conversations with classmates I do not really know.”

The lack of casual interactions in hallways, study groups, or campus events contributed to feelings of alienation. Another student shared, “My performance has definitely suffered as a result of not feeling like part of a learning community.” These accounts suggest that fostering a sense of belonging and peer support is critical for both academic and emotional resilience in medical education.

#### Mental health

Mental health challenges were reported by 19% (*n* = 11) of respondents, with students describing increased feelings of anxiety, loneliness, and emotional exhaustion. One participant candidly stated, “COVID-19 has been a nightmare for my education and mental health. The social isolation is crushing.” Another added, “It’s been laughable how neutered my experience has been. I have not met more than 20 people from my class.”

Interestingly, a few students described personality traits or behavioral adaptations that helped them maintain mental well-being during this period. For example, one student said, “My mental health has been fine, probably only because I’ve kept a relatively steady workout routine.” These findings suggest that while mental health concerns were widespread, individual coping mechanisms played a pivotal role in mitigating emotional distress.

#### Convenience

In contrast to the dominant themes of difficulty and distress, 7% (*n* = 4) of respondents highlighted benefits of the virtual format. These students appreciated the efficiency and flexibility afforded by online learning. One participant noted, “I dislike not meeting people, but I feel much more efficient because travel and preparation time have been cut down significantly.” Another added, “Overall, I’ve enjoyed virtual school because of the flexibility in my schedule.” While this theme was less prevalent, it highlights an important counterpoint: the remote learning environment may better align with certain personality types or learning preferences.

## Discussion

The COVID-19 pandemic disrupted medical education globally, magnifying systemic inequalities in access, engagement, and emotional well-being. This study revealed significant psychosocial, cognitive, and emotional impacts on first-year medical students, underscoring the broader challenges posed by crisis-driven transitions to digital education. These findings align with global discussions on the right to education, particularly in addressing inequalities exacerbated by digitalization, privatization, and limited institutional preparedness during crisis situations (15).

### Inequities in digital access and educational outcomes

Digital access emerged as a critical determinant of student engagement and academic success. The results highlighted difficulties with screen fatigue, focus, and the lack of separation between academic and personal spaces, leading to diminished motivation and reduced accountability in virtual learning environments (Difficulty Engaging, 31.6%, *n* = 18). These findings align with broader global trends where the abrupt pivot to digital learning widened disparities, as students’ ability to participate fully often depended on pre-existing resources, including access to reliable internet, private study spaces, and supportive home environments (16–18).

Isolation was another dominant theme, with 89.5% (*n* = 51) of respondents describing profound social and emotional disconnection. Peer relationships, critical for academic collaboration and emotional support, were largely disrupted. Students reported that the absence of informal, unstructured interactions made it difficult to form connections or spontaneously exchange knowledge with classmates. Many students expressed that virtual platforms, while useful for formal communication, failed to replicate the ease of casual conversation or relationship-building. This reflects a larger global issue, where digital platforms often fail to replicate the relational aspects of traditional learning environments (19).

These findings suggest that while digital platforms provided a necessary bridge during the crisis, they remain insufficient substitutes for the social and emotional dimensions of in-person education. Hybrid models that combine virtual flexibility with intentional in-person opportunities for peer interaction may provide a more balanced approach to addressing these inequities (16). While some students in other settings may have participated in virtual social events—such as Zoom hangouts or meals—this type of engagement was not described in our dataset. The survey did not explicitly probe for reasons behind the lack of virtual peer interaction, representing an area where further qualitative inquiry is warranted.

### Institutional support mechanisms and mental health services

The results emphasize the importance of sustained institutional investment in student mental health and well-being during crisis situations. Mental health concerns were prevalent, with 19% (*n* = 11) of respondents reporting heightened anxiety, loneliness, and emotional exhaustion. Students described the emotional toll of prolonged isolation, often compounded by academic stress and the absence of social support networks. These findings are consistent with global research indicating that students in crisis situations face compounded mental health risks when institutional resources are insufficient (20, 21). However, a subset of students described protective factors that supported their well-being, such as maintaining consistent routines, prioritizing physical activity, or leveraging pre-existing social networks. These strategies contributed to their emotional resilience during the disruption. This suggests that resilience-building interventions should be integrated into educational frameworks as proactive measures rather than reactive responses.

Institutions must ensure accessible and well-publicized mental health resources, including counseling services, peer support programs, and structured wellness initiatives tailored to address the unique emotional and academic stressors of medical students. Beyond crisis response, these resources should be embedded as core elements of medical education systems to safeguard student well-being (22).

### Privatization and dependency on digital platforms

The pandemic highlighted the growing reliance on privatized educational technologies and platforms, which often come with financial and accessibility barriers. Although a minority of students (7%, *n* = 4) appreciated the convenience and flexibility of virtual learning, the broader reliance on commercial digital platforms raises concerns about equity and transparency. For students who lack access to these technologies, educational inequalities are further amplified (17, 18).

Privatization in education, while offering innovative solutions, often prioritizes profit over equity. As institutions become more dependent on proprietary digital tools, the right to equitable and accessible education risks being undermined, especially during crises when rapid adoption of these platforms becomes essential. Addressing these challenges may require additional institutional oversight, transparent agreements with private vendors, and mechanisms to ensure that digital tools do not exacerbate existing inequalities (17, 23).

### Implications for the right to education in crisis situations

This study demonstrates how crisis-driven digital transitions have exposed and intensified pre-existing educational inequities. Access to quality education, especially in professional training programs like medicine, is not merely a logistical challenge—it is a fundamental human rights issue (15). The findings reinforce the importance of viewing education not as a privilege but as an essential right, one that must be safeguarded through deliberate institutional and policy-level interventions.

Medical schools must adopt a student-centered approach to crisis preparedness, prioritizing equitable access to digital tools, mental health resources, and opportunities for peer engagement. Student feedback mechanisms, such as anonymous surveys and focus groups, should remain central to institutional decision-making processes to ensure that adaptations remain responsive to evolving student needs (24, 25).

### Addressing structural inequalities through policy and practice

Structural inequalities in education extend beyond crisis periods, but crises often serve to magnify and expose these vulnerabilities. The results revealed stark disparities in how students experienced isolation, academic disengagement, and mental health challenges, depending on their pre-existing resources and support systems. Addressing these structural barriers requires long-term, equity-focused strategies, including:

- Investment in Digital Infrastructure: Ensuring all students have reliable internet access, appropriate devices, and supportive learning spaces.
- Prioritizing Mental Health Services: Embedding counseling, peer support, and wellness initiatives into standard medical education curricula.
- Hybrid Learning Models: Balancing the logistical efficiency of digital platforms with the relational and community-building benefits of in-person interactions.
- Transparent Use of Educational Technologies: Reducing reliance on privatized tools that risk excluding marginalized student populations.

These measures align with global recommendations for safeguarding the right to education during crises, emphasizing equity, resilience, and student-centered policy design (17, 22, 25, 26).

### Limitations

While this study provides valuable insights into the psychosocial, academic, and emotional impacts of the COVID-19 pandemic on first-year medical students, several limitations must be considered when interpreting the findings. First, the study relied on self-reported qualitative survey responses, which are inherently subjective and prone to biases such as selective memory recall and social desirability. Students’ perceptions, while rich in detail, may not fully capture objective measures of their mental health, academic performance, or social engagement during the pandemic. Additionally, the response rate was approximately 36% of the eligible student cohort. While sufficient for qualitative analysis, this limits the generalizability of the findings, as non-responders may have had different experiences and challenges that remain unrepresented in this study.

Another key limitation stems from the study’s single-institution context. The institutional policies, available resources, and unique local responses to the pandemic likely shaped students’ experiences in ways that may not reflect broader trends across medical schools. Similarly, the absence of quantitative mental health or academic performance measures prevents a more standardized comparison with pre-pandemic cohorts or other studies employing validated tools. This reliance on qualitative methods, while insightful, limits the ability to quantify the prevalence or intensity of specific challenges reported by students.

Furthermore, the study’s reliance on secondary data analysis introduced additional constraints. The survey was originally designed as part of an evaluation of a Continuing Medical Education (CME) intervention and was not tailored explicitly to explore the nuanced psychosocial dimensions of transitioning to medical school during a global health crisis. As such, the scope of the data was shaped by the original survey objectives, potentially leaving certain dimensions of students’ experiences underexplored.

The time-bound nature of the study also poses challenges for broader applicability. Data were collected during the 2020–2021 academic year—a period marked by rapid changes in public health guidelines and institutional responses. As these conditions shifted over time, so too did the experiences and needs of students, making it difficult to extrapolate findings to other phases of the pandemic or future crises. Additionally, the limited socioeconomic representation within the sample is notable. With only two respondents identifying as Pell Grant-eligible, the experiences of students from low-income backgrounds—a group likely to face heightened barriers to virtual learning and academic engagement—are underrepresented in this analysis.

Another limitation of the survey data was the absence of targeted questions regarding efforts students may have made to maintain social engagement through virtual platforms. While participants frequently described feelings of isolation and disconnection, we did not ask whether students had access to or participated in virtual social events. As a result, we were unable to explore potential facilitators or barriers to informal peer interaction in digital formats.

Finally, the cross-sectional nature of this study captures student experiences at a single point in time, providing a static snapshot of their challenges and coping mechanisms. A longitudinal study design would have offered a more dynamic perspective, allowing for an understanding of how students’ academic, emotional, and social experiences evolved throughout the pandemic.

Despite these limitations, the results of this study offer critical insights into the intersection of crisis-driven digitalization, structural inequities, and student well-being. It serves as an important foundation for future research and institutional policy changes aimed at fostering more resilient, equitable, and student-centered educational systems in the face of ongoing and future crises.

### Future research directions

This study highlights critical gaps for future research. Longitudinal studies tracking pandemic-era cohorts through their subsequent years of medical education may provide deeper insights into the long-term impacts of crisis-driven transitions on professional identity, mental health, and academic engagement. Additionally, research exploring how specific personality traits, coping mechanisms, and social support systems interact with crisis-related stressors could inform targeted interventions for vulnerable student populations. Future studies should also examine why certain virtual engagement strategies—such as informal Zoom-based social events—were or were not adopted, and how these shaped students’ sense of community during remote learning.

## Conclusion

The COVID-19 pandemic illuminated the fragility of education systems and their reliance on digital platforms during crises. This study provides insight into the academic, emotional, and social challenges experienced by first-year medical students during this period, emphasizing the importance of addressing digital access disparities, social isolation, and mental health vulnerabilities. Ensuring equitable access to education requires sustained institutional investment, hybrid educational models, and transparent governance of educational technologies. Above all, educational policies must remain anchored in the principles of equity, inclusivity, and the fundamental right to education, even in times of crisis (15, 17, 25).

## Data Availability

The original contributions presented in the study are included in the article/supplementary material, further inquiries can be directed to the corresponding author.
